# Liquid chromatography coupled to high-resolution mass spectrometry metabolomics: A useful tool for investigating tumor secretome based on a three-dimensional co-culture model

**DOI:** 10.1371/journal.pone.0274623

**Published:** 2022-09-21

**Authors:** Andrea C. Pelosi, Anna Maria A. P. Fernandes, Leonardo F. Maciel, Alex A. R. Silva, Giulia C. Mendes, Luísa F. Bueno, Lívia Maria F. Silva, Rafael F. Bredariol, Maycon G. Santana, Andreia M. Porcari, Denise G. Priolli

**Affiliations:** 1 Health Sciences Postgraduate Program, São Francisco University, Bragança Paulista, São Paulo, Brazil; 2 MS4Life Laboratory of Mass Spectrometry, Health Sciences Postgraduate Program, São Francisco University, Bragança Paulista, São Paulo, Brazil; 3 Multidisciplinary Laboratory, Medical School, Sao Francisco University, Bragança Paulista, São Paulo, Brazil; 4 Multiprofessional Nursing Residency Program in Oncology, A.C. Camargo Cancer Center, São Paulo, Brazil; University of Pisa, ITALY

## Abstract

Three-dimensional (3D) cell culture technologies, which more closely mimic the complex microenvironment of tissue, are being increasingly evaluated as a tool for the preclinical screening of clinically promising new molecules, and studying of tissue metabolism. Studies of metabolites released into the extracellular space (secretome) allow understanding the metabolic dynamics of tissues and changes caused by therapeutic interventions. Although quite advanced in the field of proteomics, studies on the secretome of low molecular weight metabolites (< 1500 Da) are still very scarce. We present an untargeted metabolomic protocol based on the hybrid technique of liquid chromatography coupled with high-resolution mass spectrometry for the analysis of low-molecular-weight metabolites released into the culture medium by 3D cultures and co-culture (secretome model). For that we analyzed HT-29 human colon carcinoma cells and 3T3-L1 preadipocytes in 3D-monoculture and 3D-co-culture. The putative identification of the metabolites indicated a sort of metabolites, among them arachidonic acid, glyceric acid, docosapentaenoic acid and beta-Alanine which are related to cancer and obesity. This protocol represents a possibility to list metabolites released in the extracellular environment in a comprehensive and untargeted manner, opening the way for the generation of metabolic hypotheses that will certainly contribute to the understanding of tissue metabolism, tissue-tissue interactions, and metabolic responses to the most varied interventions. Moreover, it brings the potential to determine novel pathways and accurately identify biomarkers in cancer and other diseases. The metabolites indicated in our study have a close relationship with the tumor microenvironment in accordance with the literature review.

## Introduction

Colorectal cancer (CRC) is the third most common cancer in the world and the second most deadly. Every year 1,8 million people are diagnosed and about 900,000 patients die from CRC [1]. It is usually diagnosed at advanced stages due to the limitations of current screening methods used in the clinic [2–4]. Only two blood-based biomarkers are available to monitor CRC patients: carcinoembryonic antigen (CEA) and carbohydrate antigen 19–9 (CA19-9). CEA, is a high molecular weight glycoprotein, found in embryonic tissue and colorectal malignancies. However, high levels of this compound in the blood are not specific for CRC and elevated levels of CEA are found in advanced stages of a fraction of CRC patients. The CA19-9 antigen, compared to CEA, is less sensitive and specific for CRC [5]. There is an urgent need to develop new biomarkers and modalities to detect, diagnose, and monitor the disease.

Pre-clinical *in vitro* evaluation is traditionally carried out in two-dimensional (2D) cell monoculture representing an easy and well-established methodology. The growth in 2D surface results in cell and cytoskeleton’s flattening and remodeling, changing important factors in the tumor microenvironment *in vivo* such as nuclear form, protein and lipid synthesis, biochemical responses, and signaling cascades. It is widely held that 2D culture is unable to simulate the original tumor microenvironment, which grows three-dimensionally (3D) [6, 7]. This is why many compounds and drugs are active in 2D culture models but are not successful in subsequent preclinical tests [8, 9].

The 3D culture systems have received attention to avoid certain disadvantages of 2D-culture models [10, 11]. Three-D spheroids are formed by cell aggregation mediated by the interaction between integrin and extracellular matrix with subsequent compaction by transmembrane protein interactions such as E-cadherin [12, 13]. This allows three-dimensional cell cultures to structure similar to natural tissues, to present intercellular interactions and adhesions, and simulate *in vivo* tumor characteristics such as hypoxia, necrosis, invasion, metastasis, anti-apoptosis and drug resistance [14–18].

Metabolomics, an approach targeted at comprehensive profiling of the metabolites in a biological system, has demonstrated its great potential for use in the early diagnosis and personalized treatment of various cancers including CRC [19, 20]. By applying advanced analytical techniques and bioinformatics tools, the metabolome can be mined for biomarkers associated with carcinogenesis and prognosis [4].

The metabolome is the set of molecules below 1.5 kDa produced in response to intrinsic biological and environmental factors [21]. It is the net result of the integration of systemic metabolic processes and reveals the metabolite-enzyme relationships that regulate these processes. Thus, the metabolome, in contrast to genome or proteome, has been considered a more instantaneous representation of the phenotype, since its changes occur more quickly than changes in genes or proteins, and may indicate, in a time closer to the real one, current biological events. Additionally, several drug target a specific metabolite by inhibiting its enzyme or receptor, which are proteins and not genetic sequences. In this context, metabolomics studies seem more promising for discovering new molecules and metabolic pathways for potential therapeutic targets. Thus, recognizing that metabolites play significant and dynamic roles in biological processes has made metabolomics a key area in studies of systemic profiles in diseases and medicine in general [22].

Metabolomic studies that use liquid chromatography coupled to mass spectrometry (LC-MS) as an analytical technique, provide a comprehensive analysis of the metabolome and revolutionize the study of small molecules. LC-MS-based metabolomics can be categorized into (i) targeted analysis which is a pre-established quantitative analytical approach to a list of known metabolites and (ii) untargeted metabolomics which is characterized by the simultaneous measurement of a large number of metabolites of each sample, usually without prior knowledge of the constituents and changes in them. The main advantage of untargeted metabolomics is the discovery of new metabolites in relation to the study context; therefore, it is considered a hypothesis-generating approach [23].

Two approaches are considered when performing metabolomic studies of cultured cell lines. Those focused on intracellular metabolites of isolated cells and those focused on the secretome or extracellular metabolites released (ERM) by cells in the culture medium. The analysis of ERM provides a picture of the metabolites resulting from the exchange carried out between the cells and the culture medium. This approach has the following advantages: ensuring little (or non-existent) handling of cells, which avoids the production of artifacts, allowing the monitoring of metabolic activity in response to experimental disturbances without cell disruption, enabling the monitoring of metabolic changes over time within the same culture and avoid carrying out long and multiple extraction procedures, which also enable the production of technical artifacts that can lead to concealment or unwanted manipulation of biological results [24]. However, the disadvantages of working with the secretome include matrix effects related to the salty media composition, which is rich in sugars, lipids, proteins, and water and might interfere in the analysis. The dilution of the metabolites of interest in the media also impacts the detection sensitivity [25].

Here we present a liquid chromatography coupled with high-resolution mass spectrometry metabolomic based protocol for the analyses of ERM. For the development of the protocol, culture medium from monoculture spheroids and co-culture (from HT-29 and 3T3-L1 cells) were analyzed. The putative identification of relevant molecular features for each spheroid type and those influenced by co-culture demonstrate the applicability of the method to the study of the metabolism of these spheroids and their tissue-tissue interactions with a focus on discovering new therapeutic targets, biomarkers and their associated metabolic pathways.

## Material and methods

The protocol described in this peer-reviewed article is published on protocols.io, [doi.org/10.17504/protocols.io.b24vqgw6] and is included for printing as S1 File with this article.

### Expected results

For this protocol, we used untargeted ultra-performance liquid chromatography coupled to electrospray ionization quadrupole time-of-flight mass spectrometry (UPLC-QTOF) operating in high energy collision spectral acquisition mode (MS^E^) mode approach to investigate differences between extracellular metabolomic profiles of HT-29 and those of 3T3-L1 spheroids. The presence of adipose tissue can influence the development of CRC in vivo [26]. To better understand this influence in vitro, we used a model of HT-29 cell line as cancer cells and 3T3-L1 as adipocytes [27]. These cell lines were cultured separately and co-cultured, and their secretome was used to investigate the tumor microenvironment and the interaction between cancer cells and the adipocyte tissue.

CSH (charged surface hybrid) particles were designed to enable sample loading and improve peak symmetry when using low ionic strength mobile phases, as instructed by the manufacturer. Reversed-phase columns, such as the chosen CSH C18, are broadly used for metabolomics investigation in different matrices such as plasma [20], serum [28], urine [29], tissue [30], and cell cultures [31], as chromatographic columns of this type generally result in the detection of more features [32]. The ERM obtained after the co-cultivation of both cell types was investigated. In the beginning, a total of 2658 molecular features were detected in the positive ionization mode and 3521 features were detected in the negative ionization mode. These features retain the information of retention time and mass-to-charge ratio (tR_*m/z*) of each metabolite elucidated after the LC-MS runs.

Volcano plot statistical analysis of all cultured conditions compared with the blank samples (cultured medium only) provide the number of characteristic metabolites of each spheroid as reported in Table 1.

**Table 1:** Molecular features of 3D cultured cells.

Cell Type	Negative Mode	Positive Mode
HT-29	191 features	3 features
3T3-L1	329 features	267 features
HT-29 + 3T3-L1	129 features	14 features

a. characteristic metabolites of each spheroid after comparison with the blank samples (cultured media only).

Only molecular features with log2 (FC) > 0 were considered because they represent an increase of the metabolite abundance in the medium due to the contact with the organoids. The statistical comparison (volcano plot) of all three different conditions was also performed and returned some relevant metabolites of each cell type as depicted in Table 2. Although Table 2 brings a small number of metabolites when compared to the detected ones, these metabolites were the ones that met the statistical criteria of relevance, as well as the annotation criteria based on MS/MS and isotopic pattern recognition. Indeed, in untargeted metabolomics, linking chemical structures to the data obtained by mass spectrometry remains a significant challenge. The vast majority of information collected by metabolomics is the so-called "dark matter," i.e., chemical signatures that remain uncharacterized [33].

**Table 2:** Representative secretome from 3D culture or 3D co-cultured spheroids.

Feature Code^a^	Log2 (FC)^b^	Putative assignment	Identifiers^c^		Comparative abundances^d^		
^tR^_m/z					HT-29	3T3-L1	HT-29 + 3T3-L1
8.49_303.2320m/z	1.5	Arachidonic Acid	C00219	HMDB0001043	✓	NA	DOWN
0.75_151.0247m/z	3.2	Glyceric Acid	C00258	HMDB0000139	✓	DOWN	DOWN
8.59_329.2473m/z	1.1	Docosapentaenoic Acid*	C16513	HMDB0001976	✓	DOWN	DOWN
0.61_134.0460m/z	-4.0	Beta-Alanine	C00099	HMDB0000056	✓	DOWN	UP
1.67_291.0973m/z	9.3	γ-Glutamyltyrosine	C03363	HMDB0011741	✓	NA	NS
3.52_241.1184m/z	6.8	γ-Glutamylisoleucine	C03363	HMDB0011170	✓	NA	NS
8.13_301.2162m/z	3.5	5-HETE	C04805	HMDB0011134	✓	NA	NS
9.00_305.2475m/z	2.5	Dihomo-γ-linolenic acid	C03242	HMDB0002925	✓	NA	NS
0.77_153.0402m/z	4.8	Xanthine	C00385	HMDB0000292	✓	NA	NS
0.54_139.0743n	1.5	Histidinal	C01929	HMDB0012234	NA	NA	✓
0.56_251.1008n	2.6	Deoxyadenosine	C00559	HMDB0000101	NA	NA	✓
4.58_245.0920m/z	15.5	Formyl-N-acetyl-5-methoxykynurenamine	C05642	HMDB0004259	NA	✓	NS
0.54_802.6697m/z	11.5	PC(o-38:0)	C00958	HMDB0013408	NA	✓	NS
0.61_232.0824m/z	8.4	2-Keto-6-acetamidocaproate	C05548	HMDB0012150	NA	✓	NS
4.43_407.1214m/z	7.9	2-S-glutathionyl acetate	C14862	HMDB0062198	NA	✓	NS
1.39_298.0970m/z	7.7	5’-Methylthioadenosine	C00170	HMDB0001173	NA	✓	NS
0.65_152.0566m/z	7.6	Guanine	C00242	HMDB0000132	NA	✓	NS
0.50_364.2445m/z	7.0	MAG(14:1)	C01885	HMDB0011531	NA	✓	NS

a. tR = retention time; m/z = mass-to-charge ratio.

b. Compared to blank samples (culture media only).

c. HMDBXXXXXXX, metabolites described in the Human Metabolome Database (HMDB—https://hmdb.ca/); CXXXXX, described in the Kyoto Encyclopedia of Genes and Genomes database (KEGG—https://www.genome.jp/kegg/).

d. ✓ = presence; NA = absence; UP and DOWN = more or less abundant respectively, when compared with HT-29 culture; NS = not significantly impacted when compared with HT-29 culture.

* Annotated by exact mass only.

**Fig 1:**
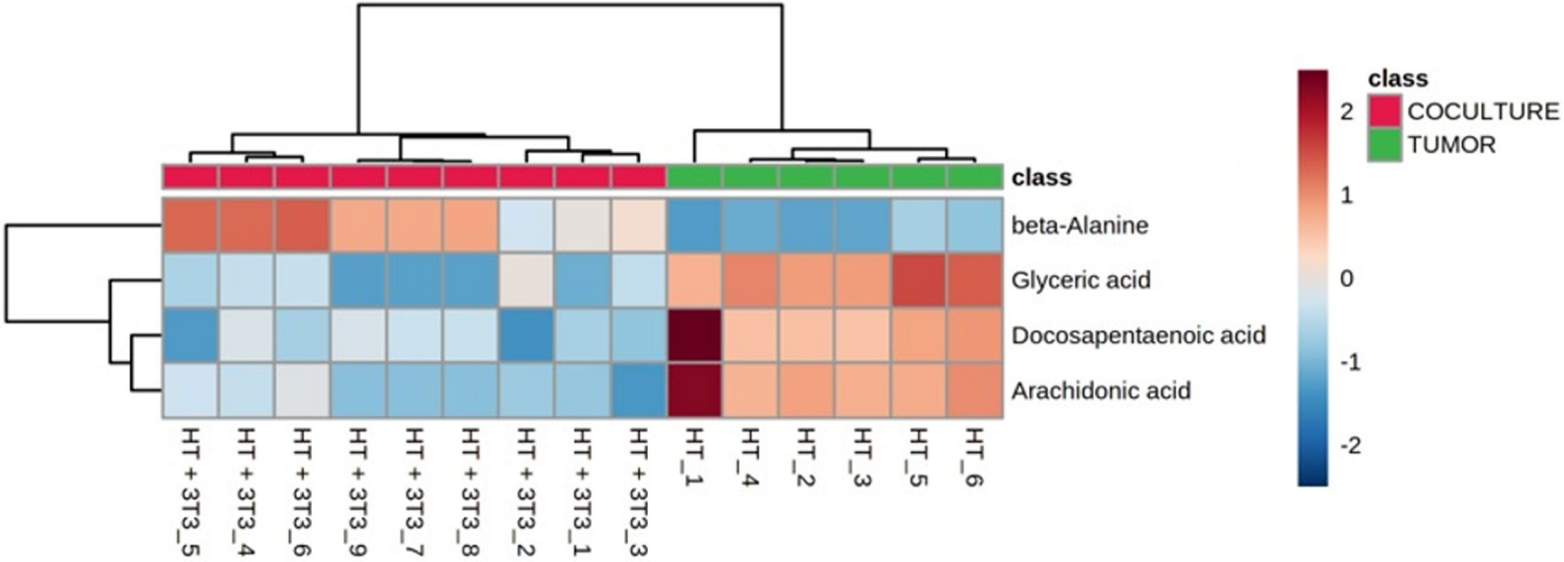
Heatmap of the metabolites from HT spheroids impacted by the co-culture with 3T3 adipocytes. The colors are normalized to the relative abundance of each metabolite. Samples (vertical axis) and metabolites (horizontal axis) are separated by Ward’s algorithm and the dendrogram was scaled using Pearson’s correlation. The clusters containing tumor spheroid alone and co-culture with tumor and adipocyte spheroids are highlighted in green and red, respectively.

Arachidonic acid (ARA), 5-HETE, and dihomo-γ-linolenic acid (DGLA) stood out among the listed metabolites. Studies have related ARA in the colorectal cancer carcinogenesis process, with the influence of the inflammatory process on tumor growth and progression through the interaction of inflammatory cytokines and chemokines with tumor cells [34–36]. Cajal cells and F2d fibroblasts, mesenchymal components of colonic tissue related to CCR, have already demonstrated high concentrations of ARA metabolism genes [37, 38]. Therefore, ARA was studied as a therapeutic target due to its direct involvement in the process of inflammation and carcinogenesis of colorectal cancer. Corroborating to these data, the use of ibuprofen and aspirin by adult patients reduced the risk of progressing to cancer for premalignant and advanced-stage lesions, as well as for recurrent adenomas [39, 40], suggesting that ARA inhibition may indeed play an important role in colorectal carcinogenesis. Fig 1 shows the increase of some metabolites, ARA included, which were positively or negatively impacted by the CRC spheroid in the presence of adipocytes. Furthermore, studies demonstrate the increase of 5-lipoxygenase (5-LOX), part of the ARA pathway, and its metabolite, 5-HETE, in tumor tissues of the prostate, pancreas, colon, stomach and cervix [41–46].

DGLA is related to linoleic acid metabolism, an unsaturated fatty acid found in omega-6, which is associated with increased tumor growth, size, and metastatic potential [47]. Diets rich in omega-6 would be related to pro-inflammatory effects in the body, which may predispose to CRC development in long-term exposure [47, 48]. Also noteworthy are γ-glutamyltyrosine and γ-glutamylisoleucine as glutathione metabolites, a potential biomarker of tumorigenesis, beta-alanine, an indicator of tumor protein metabolism reprogramming, and histidinal, a metabolite of histidine, which is involved in several biological responses related to tumor growth [49–52]. The biological findings point to relevant aspects of tumor metabolism and highlight the potential of the protocol described here as a valuable tool in the metabolism study of *in vitro* model CCR based on 3D culture and co-culture cells.

## Supporting information

S1 File Protocol to secretome investigation of tumor 3D co-culture model. (PDF)

## References

[1] International Agency for Research on Cancer. Globocan 2018: Cancer Fact Sheets—Colorectal Cancer. IARC http://gco.iarc.fr/today/data/factsheets/cancers/10_8_9-Colorectum-fact-sheet.pdf (2018).

[2] Grabenbauer GG, Holger G. Management of radiation and chemotherapy related acute toxicity in gastrointestinal cancer. Best Pract Res Clin Gastroenterol. 2016;30(4):655–64. doi: 10.1016/j.bpg.2016.06.001 27644912

[3] Berger AM, Grem JL, Visovsky C, Marunda HA, Yurkovich JM. Fatigue and other variables during adjuvant chemotherapy for colon and rectal cancer. Oncol Nurs Forum. 2010;37(6):E359–69. doi: 10.1188/10.ONF.E359-E369 21059569

[4] Ni Y, Xie G, Jia W. Metabonomics of human colorectal cancer: new approaches for early diagnosis and biomarker discovery. J Proteome Res. 2014;13(9):3857–70. doi: 10.1021/pr500443c 25105552

[5] Gonzalez-Pons M, Cruz-Correa M. Colorectal Cancer Biomarkers: Where Are We Now? Biomed Res Int. 2015;2015:149014. doi: 10.1155/2015/149014 26106599 PMC4461726

[6] Souza AG, Silva IBB, Campos-Fernandez E, Barcelos LS, Souza JB, Marangoni K, et al. Comparative Assay of 2D and 3D Cell Culture Models: Proliferation, Gene Expression and Anticancer Drug Response. Curr Pharm Des. 2018;24(15):1689–94. doi: 10.2174/1381612824666180404152304 29623827

[7] Knight E, Przyborski S. Advances in 3D cell culture technologies enabling tissue-like structures to be created in vitro. J Anat. 2015;227(6):746–56. doi: 10.1111/joa.12257 25411113 PMC4694114

[8] Wilding JL, Bodmer WF. Cancer cell lines for drug discovery and development. Cancer Res. 2014;74(9):2377–84. doi: 10.1158/0008-5472.CAN-13-2971 24717177

[9] Kapałczyńska M, Kolenda T, Przybyła W, Zajączkowska M, Teresiak A, Filas V, et al. 2D and 3D cell cultures—a comparison of different types of cancer cell cultures. Archives of medical science: AMS. 2018;14(4):910–9. doi: 10.5114/aoms.2016.63743 30002710 PMC6040128

[10] Azzarelli R. Organoid Models of Glioblastoma to Study Brain Tumor Stem Cells. Frontiers in cell and developmental biology. 2020;8:220-. doi: 10.3389/fcell.2020.00220 32373607 PMC7176979

[11] Liu Q, Yang C, Wang S, Shi D, Wei C, Song J, et al. Wnt5a-induced M2 polarization of tumor-associated macrophages via IL-10 promotes colorectal cancer progression. Cell Commun Signal. 2020;18(1):51. doi: 10.1186/s12964-020-00557-2 32228612 PMC7106599

[12] Drost J, Clevers H. Organoids in cancer research. Nat Rev Cancer. 2018;18(7):407–18. doi: 10.1038/s41568-018-0007-6 29692415

[13] Lin RZ, Chang HY. Recent advances in three-dimensional multicellular spheroid culture for biomedical research. Biotechnol J. 2008;3(9–10):1172–84. doi: 10.1002/biot.200700228 18566957

[14] Altunbek M, Çetin D, Suludere Z, Çulha M. Surface-enhanced Raman spectroscopy based 3D spheroid culture for drug discovery studies. Talanta. 2019;191:390–9. doi: 10.1016/j.talanta.2018.08.087 30262075

[15] Benien P, Swami A. 3D tumor models: history, advances and future perspectives. Future Oncol. 2014;10(7):1311–27. doi: 10.2217/fon.13.274 24947267

[16] Breslin S O’Driscoll L. Three-dimensional cell culture: the missing link in drug discovery. Drug Discov Today. 2013;18(5–6):240–9. 23073387 10.1016/j.drudis.2012.10.003

[17] Lee JY, Chaudhuri O. Modeling the tumor immune microenvironment for drug discovery using 3D culture. APL Bioeng. 2021;5(1):010903. doi: 10.1063/5.0030693 33564739 PMC7857858

[18] Meier-Hubberten JC, Sanderson MP. Establishment and Analysis of a 3D Co-Culture Spheroid Model of Pancreatic Adenocarcinoma for Application in Drug Discovery. Methods Mol Biol. 2019;1953:163–79. doi: 10.1007/978-1-4939-9145-7_11 30912022

[19] Messias MCF, Mecatti GC, Priolli DG, de Oliveira Carvalho P. Plasmalogen lipids: functional mechanism and their involvement in gastrointestinal cancer. Lipids in Health and Disease. 2018;17(1):41. doi: 10.1186/s12944-018-0685-9 29514688 PMC5842581

[20] Silva AAR, Cardoso MR, Rezende LM, Lin JQ, Guimaraes F, Silva GRP, et al. Multiplatform Investigation of Plasma and Tissue Lipid Signatures of Breast Cancer Using Mass Spectrometry Tools. Int J Mol Sci. 2020;21(10). doi: 10.3390/ijms21103611 32443844 PMC7279467

[21] Nicholson JK, Lindon JC. Systems biology: Metabonomics. Nature. 455. England 2008. p. 1054–6. doi: 10.1038/4551054a 18948945

[22] Cui L, Lu H, Lee YH. Challenges and emergent solutions for LC-MS/MS based untargeted metabolomics in diseases. Mass Spectrom Rev. 2018;37(6):772–92. doi: 10.1002/mas.21562 29486047

[23] Nalbantoglu S. Metabolomics: Basic Principles and Strategies. In: Nalbantoglu S., Amri H., editors. Molecular Medicine [Internet].

[24] Campanella B, Colombaioni L, Nieri R, Benedetti E, Onor M, Bramanti E. Unraveling the Extracellular Metabolism of Immortalized Hippocampal Neurons Under Normal Growth Conditions. Frontiers in chemistry. 2021;9:621548-. doi: 10.3389/fchem.2021.621548 33937186 PMC8085660

[25] Pinu FR, Villas-Boas SG. Extracellular Microbial Metabolomics: The State of the Art. Metabolites. 2017;7(3). doi: 10.3390/metabo7030043 28829385 PMC5618328

[26] Jochem C, Leitzmann M. Obesity and Colorectal Cancer. Recent Results Cancer Res. 2016;208:17–41. doi: 10.1007/978-3-319-42542-9_2 27909900

[27] Zebisch K, Voigt V, Wabitsch M, Brandsch M. Protocol for effective differentiation of 3T3-L1 cells to adipocytes. Anal Biochem. 2012;425(1):88–90. doi: 10.1016/j.ab.2012.03.005 22425542

[28] Zardini Buzatto A, Tatlay J, Bajwa B, Mung D, Camicioli R, Dixon RA, et al. Comprehensive Serum Lipidomics for Detecting Incipient Dementia in Parkinson’s Disease. J Proteome Res. 2021;20(8):4053–67. doi: 10.1021/acs.jproteome.1c00374 34251208

[29] Struck-Lewicka W, Kordalewska M, Bujak R, Yumba Mpanga A, Markuszewski M, Jacyna J, et al. Urine metabolic fingerprinting using LC-MS and GC-MS reveals metabolite changes in prostate cancer: A pilot study. J Pharm Biomed Anal. 2015;111:351–61. doi: 10.1016/j.jpba.2014.12.026 25684700

[30] Al-Sari N, Suvitaival T, Mattila I, Ali A, Ahonen L, Trost K, et al. Lipidomics of human adipose tissue reveals diversity between body areas. PLoS One. 2020;15(6):e0228521. doi: 10.1371/journal.pone.0228521 32544198 PMC7297320

[31] Ali AS, Raju R, Ray S, Kshirsagar R, Gilbert A, Zang L, et al. Lipidomics of CHO Cell Bioprocessing: Relation to Cell Growth and Specific Productivity of a Monoclonal Antibody. Biotechnol J. 2018;13(10):e1700745. doi: 10.1002/biot.201700745 29521466

[32] Yanes O, Tautenhahn R, Patti GJ, Siuzdak G. Expanding coverage of the metabolome for global metabolite profiling. Anal Chem. 2011;83(6):2152–61. doi: 10.1021/ac102981k 21329365 PMC3285547

[33] Silva RR, Dorrestein PC, Quinn RA. Illuminating the dark matter in metabolomics. Proc Natl Acad Sci U S A. 2015;112(41):12549–50. doi: 10.1073/pnas.1516878112 26430243 PMC4611607

[34] Mizuno R, Kawada K, Itatani Y, Ogawa R, Kiyasu Y, Sakai Y. The Role of Tumor-Associated Neutrophils in Colorectal Cancer. Int J Mol Sci. 2019;20(3). doi: 10.3390/ijms20030529 30691207 PMC6386937

[35] Mármol I, Sánchez-de-Diego C, Pradilla Dieste A, Cerrada E, Rodriguez Yoldi MJ. Colorectal Carcinoma: A General Overview and Future Perspectives in Colorectal Cancer. Int J Mol Sci. 2017;18(1). doi: 10.3390/ijms18010197 28106826 PMC5297828

[36] Yang Y, Tang LQ, Wei W. Prostanoids receptors signaling in different diseases/cancers progression. J Recept Signal Transduct Res. 2013;33(1):14–27. doi: 10.3109/10799893.2012.752003 23327583

[37] Roulis M, Kaklamanos A, Schernthanner M, Bielecki P, Zhao J, Kaffe E, et al. Paracrine orchestration of intestinal tumorigenesis by a mesenchymal niche. Nature. 2020;580(7804):524–9. doi: 10.1038/s41586-020-2166-3 32322056 PMC7490650

[38] Smyth EM, Grosser T, Wang M, Yu Y, FitzGerald GA. Prostanoids in health and disease. J Lipid Res. 2009;50 Suppl(Suppl):S423–8. doi: 10.1194/jlr.R800094-JLR200 19095631 PMC2674745

[39] Chudy-Onwugaje K, Huang WY, Su LJ, Purdue MP, Johnson CC, Wang L, et al. Aspirin, ibuprofen, and reduced risk of advanced colorectal adenoma incidence and recurrence and colorectal cancer in the PLCO Cancer Screening Trial. Cancer. 2021;127(17):3145–55. doi: 10.1002/cncr.33623 33974712 PMC8355096

[40] Chang J, Tang N, Fang Q, Zhu K, Liu L, Xiong X, et al. Inhibition of COX-2 and 5-LOX regulates the progression of colorectal cancer by promoting PTEN and suppressing PI3K/AKT pathway. Biochem Biophys Res Commun. 2019;517(1):1–7. doi: 10.1016/j.bbrc.2018.01.061 29339153

[41] Tang J, Zhang C, Lin J, Duan P, Long J, Zhu H. ALOX5-5-HETE promotes gastric cancer growth and alleviates chemotherapy toxicity via MEK/ERK activation. Cancer Med. 2021;10(15):5246–55. doi: 10.1002/cam4.4066 34121352 PMC8335819

[42] Li L, Xiao Y, Xu Z, Wang S. Zileuton inhibits arachidonate-5-lipoxygenase to exert antitumor effects in preclinical cervical cancer models. Cancer Chemother Pharmacol. 2021;88(6):953–60. doi: 10.1007/s00280-021-04343-w 34477945

[43] Sarveswaran S, Thamilselvan V, Brodie C, Ghosh J. Inhibition of 5-lipoxygenase triggers apoptosis in prostate cancer cells via down-regulation of protein kinase C-epsilon. Biochim Biophys Acta. 2011;1813(12):2108–17. doi: 10.1016/j.bbamcr.2011.07.015 21824498 PMC3541030

[44] Wasilewicz MP, Kołodziej B, Bojułko T, Kaczmarczyk M, Sulzyc-Bielicka V, Bielicki D, et al. Overexpression of 5-lipoxygenase in sporadic colonic adenomas and a possible new aspect of colon carcinogenesis. International journal of colorectal disease. 2010;25(9):1079–85. doi: 10.1007/s00384-010-0980-z 20549218 PMC2912725

[45] Sarveswaran S, Chakraborty D, Chitale D, Sears R, Ghosh J. Inhibition of 5-lipoxygenase selectively triggers disruption of c-Myc signaling in prostate cancer cells. The Journal of biological chemistry. 2015;290(8):4994–5006. doi: 10.1074/jbc.M114.599035 25540201 PMC4335236

[46] Hennig R, Ding X-Z, Tong W-G, Schneider MB, Standop J, Friess H, et al. 5-Lipoxygenase and leukotriene B(4) receptor are expressed in human pancreatic cancers but not in pancreatic ducts in normal tissue. The American journal of pathology. 2002;161(2):421–8. doi: 10.1016/S0002-9440(10)64198-3 12163367 PMC1850753

[47] Nixon DW. Cancer, cancer cachexia, and diet: lessons from clinical research. Nutrition. 1996;12(1 Suppl):S52–6. 8850222 10.1016/0899-9007(96)90020-9

[48] D’Angelo S, Motti ML, Meccariello R. ω-3 and ω-6 Polyunsaturated Fatty Acids, Obesity and Cancer. Nutrients. 2020;12(9):2751. doi: 10.3390/nu12092751 32927614 PMC7551151

[49] Huang J, Mondul AM, Weinstein SJ, Derkach A, Moore SC, Sampson JN, et al. Prospective serum metabolomic profiling of lethal prostate cancer. Int J Cancer. 2019;145(12):3231–43. doi: 10.1002/ijc.32218 30779128 PMC6698432

[50] Wang Y, Jacobs EJ, Carter BD, Gapstur SM, Stevens VL. Plasma Metabolomic Profiles and Risk of Advanced and Fatal Prostate Cancer. Eur Urol Oncol. 2021;4(1):56–65. doi: 10.1016/j.euo.2019.07.005 31378665

[51] Tan J, Wang HL, Yang J, Liu QQ, Li CM, Wang YQ, et al. JMJD2B-induced amino acid alterations enhance the survival of colorectal cancer cells under glucose-deprivation via autophagy. Theranostics. 2020;10(13):5763–77. doi: 10.7150/thno.38087 32483417 PMC7254993

[52] Medina VA, Rivera ES. Histamine receptors and cancer pharmacology. British journal of pharmacology. 2010;161(4):755–67. doi: 10.1111/j.1476-5381.2010.00961.x 20636392 PMC2992892

