# Supplementary material for: Liquid chromatography coupled to high-resolution mass spectrometry metabolomics: A useful tool for investigating tumor secretome based on a three-dimensional co-culture model
Source: PLoS One. 2022 Sep 21;17(9):e0274623. doi: 10.1371/journal.pone.0274623 (PMC9491614; doi:10.1371/journal.pone.0274623)
Supplement: S1 File — (PDF) [file pone.0274623.s001.pdf]

protocols.io

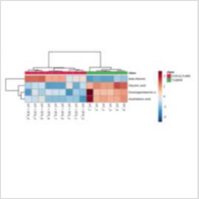

ceqmtdu6

Protocol to secretome investigation of tumor 3D co-culture model V.(ceqmtdu6)

ANNA MARIA AP FERNANDES[1], Giulia Carli Mendes[2], Alex Rosini[3], Andrea Corazzi Pelosi[1], Leonardo Maciel[2], Luísa F Bueno[2], Lívia Maria F Silva[2], Rafael F Bredariol[2], Maycon Giovani Santana[4], Andreia de Melo Porcari[3], Denise G. Priolli[1]

[1]Postgraduate Programme Stricto Sensu in Health Science, São Francisco University, Bragança Paulista, São Paulo, Brazil;

[2]Multidisciplinary Laboratory, Medical School, Sao Francisco University, Bragança Paulista, São Paulo, Brazil.;

[3]MS4Life Laboratory of Mass Spectrometry, Health Sciences Postgraduate Program, São Francisco University, Bragança Paulista, São Paulo, Brazil;

[4]Multiprofessional Nursing Residency Program in Oncology, A.C.Camargo Cancer Center, São Paulo, Brazil.

1 *Works for me*

Álex Rosini

ABSTRACT

Three-dimensional (3D) cell culture technologies, which more closely mimic the complex microenvironment of tissue, are being increasingly evaluated as a tool for the preclinical screening of clinically promising new molecules, and for the study of tissue metabolism. Studies of metabolites released into the extracellular space (secretome) allow understanding the metabolic dynamics of tissues and changes caused by therapeutic interventions. Although quite advanced in the field of proteomics, studies on the secretome of low molecular weight metabolites (< 1500 Da) are still very scarce.

We present an untargeted metabolomic protocol based on the hybrid technique of high-performance liquid-chromatography coupled with high-resolution mass spectrometry for the analysis of low-molecular-weight metabolites released into the culture medium by 3D cultures and coculture (secretoma model). For that, we analayzed HT-29 human colon carcinoma cells and 3T3-L1 preadipocytes in 3D-monoculture and 3D-coculture.

This protocol represents a possibility to list metabolites released in the extracellular environment in a comprehensive and untargeted manner, opening the way for the generation of metabolic hypotheses that will certainly contribute to the understanding of tissue metabolism, tissue-tissue interactions, and metabolic responses to the most varied interventions. Moreover, it brings potential to determine novel pathways and identify accurate biomarkers in cancer and other diseases. The metabolites indicated in our study have a close relationship with the tumor microenvironment in accordance with the literature review.

For the 3D cell culture by levitation we used Bio-Assembler™ system (Bio Science 662840, Greiner One Bio, Americana, Brazil) in the n3D Biosciences and adapted protocol published by:

- HAISLER, W. L. et al. Three-dimensional cell culturing by magnetic levitation. **Nature protocols**, v. 8, n. 10, p. 1940-1949, 2013. https://doi.org/10.1038/nprot.2013.125

PROTOCOL INFO

ANNA MARIA AP FERNANDES, Giulia Carli Mendes, Alex Rosini, Andrea Corazzi Pelosi, Leonardo Maciel, Luísa F Bueno, Lívia Maria F Silva, Rafael F Bredariol, Maycon Giovani Santana, Andreia de Melo Porcari, Denise G. Priolli . Protocol to secretome investigation of tumor 3D co-culture model. **protocols.io**
https://protocols.io/view/protocol-to-secretome-investigation-of-tumor-3d-co-ceqmtdu6
Version created by Álex Rosini

KEYWORDS

Mass Spectrometry, 3D Cell Culture, Colonic Neoplasm, Biomarkers

CREATED

Aug 02, 2022

LAST MODIFIED

Aug 19, 2022

PROTOCOL INTEGER ID

68077

GUIDELINES

- HAISLER, W. L. et al. Three-dimensional cell culturing by magnetic levitation. **Nature protocols**, v. 8, n. 10, p. 1940-1949, 2013. https://doi.org/10.1038/nprot.2013.125

MATERIALS TEXT

Trypsin EDTA **Gibco - Thermo Fischer Catalog #25-051-Cl.**

Dulbecco's Modified Eagle's Medium (DMEM) **Sigma Aldrich Catalog #D5796**

Sodium Pyruvate (100 mM) **Thermo Fisher Scientific Catalog #11360070**

Fetal Bovine Serum **Gibco - Thermo Fischer Catalog #10270106**

Gibco™ Penicillin-Streptomycin (10,000 U/mL) **Fisher Scientific Catalog #15-140-122**

Trypan Blue Solution 0.4% **Thermo Fisher Scientific Catalog #15250061**

Acetonitrile **J.T. Baker LC/MS Grade, 4 L Catalog #9829-03**

Isopropanol HPLC solvent **JT Baker Catalog #9095-02**

Water MilliQ **Contributed by users**

T25 or T75 Flask **Contributed by users**

24 Well Bio Assembler Kit **greiner bio-one Catalog #662840**

2D CELL CULTURE

1 Use human colon carcinoma (HT-29) and pre-adipocytes cells (3T3-L1) (Banco de Células do Rio de Janeiro (BCRJ; Duque de Caxias, Brazil).

2 Thaw HT-29 and 3T3-L1 cells and propagate in culture using Modified Dulbecco Eagle Medium (DMEM - Sigma D-5648, São Paulo, Brazil), supplement with [M] **100 millimolar (mM)** sodium pyruvate (Gibco -11- 360, Thermo Fisher Scientific, Waltham, Massachusetts, USA), [M] **10 % (v/v)** fetal bovine serum (Gibco 2010-09, Thermo Fisher Scientific, Waltham, Massachusetts, USA) and [M] **1 % (v/v)** antibiotics ([M] **100 U/ml** of penicillin and [M] **10 mg/mL** of streptomycin (Gibco 15140-122, Thermo Fisher Scientific, Waltham, Massachusetts, USA).

3 Culture cells in a humidified chamber with [M] **5 % (v/v)** CO_2 (HeraCELL 150) at **37 °C** .

4 Incubate cell cultures with **3 mL** trypsin-EDTA [M] **0.25 % (v/v)** (Gibco 25 200, Fisher Scientific, Waltham, Massachusetts, USA) at **37 °C** for three minutes to allow cell disaggregation and propagation.
Use DMEM plus 10% FBS to inactivate trypsin. Transfer the cell pellet to a new 75 cm^3 flask (T75) containing **10 mL** DMEM.

5 Change the culture medium according to the cell doubling time.

6 Determine cell viability in a Neubauer chamber using Trypan Blue (Gibco 15250061, Thermo Fisher Scientific, Waltham, Massachusetts, USA)

3D CELL CULTURE

7 Use the Bio-Assembler™ system (Bio Science 662840, Greiner One Bio, Americana, Brazil) in the n3D Biosciences 24-well configuration (HAISLER et al., 2013).

> Haisler WL, Timm DM, Gage JA, Tseng H, Killian TC, Souza GR (2013). Three-dimensional cell culturing by magnetic levitation.. Nature protocols.
> https://doi.org/10.1038/nprot.2013.125

8 Prepare the magnetic nanoparticles by removing it from the refrigerator and thawing it at room temperature **20-25 °C** for about 15 min.

9 Culture HT- 29 cells (passage 12th) in monolayer culture at T75 flask. Determine the cell viability (>75%) in a Neubauer chamber, when the cells confluence reaches 80%-90%.

10 Add **1 µL per 10.000 cells** of magnetic nanoparticles (Nanoshuttle™- PL, Greiner) in the single cell suspension flask, homogenize gently the suspension and centrifuge **1500 rpm, 00:05:00 , 3 times** . 5m

11 Resuspend the cells and fill each well of cell-repellent 24-well plate with an amount of solution necessary to reach 7.5 x 10^3 cells, after centrifugation.

12 Complete with **250 µL** of supplemented medium Modified Dulbecco Eagle Medium (DMEM - Sigma D-5648, São Paulo, Brazil), supplement with **100 millimolar (mM)** sodium pyruvate (Gibco -11- 360, Thermo Fisher Scientific, Waltham, Massachusetts, USA), **10 % (v/v)** fetal bovine serum (Gibco 2010-09, Fisher Scientific, Waltham, Massachusetts, USA) and **1 % (v/v)** antibiotics (**100 U/ml** of penicillin and **10 mg/mL** of streptomycin) (Gibco 15140-122, Thermo Fisher Scientific, Waltham, Massachusetts, USA) to a volume of **250 µL /well**

13 Place a magnetic coupling driver under the plate for 05:00:00 and incubate it in the humidified chamber with [M] 5 % (v/v) CO_2 (HeraCELL 150) at 37 °C . 5h

14 Close the plate and place the levitation drive atop the intermediate lid to levitate the cells.

If the cells not immediately levitate gently shake the plate moving it to back and forth, until they levitate

15 Keep the magnetic coupling drive for 7 days.

16 Use the field microscopy to verify the cohesion of the structures formed.

17 Collect the culture medium whenever the exchange is necessary. To do it, use the holding drive to hold the 3D culture down while aspirating the liquid.

18 Culture 3T3-L1 cells 600 µL in monolayer to 90% confluence and cell viability (>75%), and incubate in a supplemented medium in repellent hanging drop plate at 37 °C / [M] 5 % (v/v) CO2/ 95 % humidity and monitore until the aggregates have formed and differentiated to adipocytes.

3D CELL COCULTURE

19 Add 3T3-L1 spheroid suspension in each well of cell repellent HT-29 wells plate using a magnetic pen, 21 days after the beginning of HT-29 spheroid formation and keep it for 7 days.

EXTRACTION OF SAMPLES 10m

20 Using a pipet, take 200 µL of the culture medium of each well and place it into a microtube. Then, add 50 µL of iced isopropanol and keep the microtube at -20 °C Overnight . Prepare each sample in triplicate. Additionally, prepare a pooled quality control (QC) sample. For that, an aliquot (20 µL) of each sample must be collected and pooled together in a microtube to generate a QC sample containing the chemical composition representative of the sample set. Homogenize this sample and split it into different vials, then prepare them following the same extraction protocol. Use QC samples for system suitability before batch analysis. Also, inject a QC sample after 10 samples to investigate and filter instrumental variation. Blank samples (N=3) must also be prepared using the culture medium without adding any cell or culture and following the complete protocol for sample preparation. Samples must be organized by starting with QC samples, followed by blank samples, and then the set of test samples randomly organized. A QC sample must be placed every 10 samples. The final sample must be a QC sample.

21 Centrifuge the samples for 12880 x g, 4°C, 00:10:00 . 10m

22 Remove a medium aliquot of 150 µL of each sample and dry under nitrogen pressure.

23 Resuspend the dry extracts in 150 µL of a solution composed of p-Fluoro-DL-phenylalanine **Sigma-aldrich Catalog # F525** at [M] 200 micromolar (µM) in Methanol **Sigma Aldrich Catalog #M3641** : MilliQ water **Contributed by users** 1:1 v/v).

LIQUID CHROMATOGRAPHY ANALYSES

24 Perform chromatography analyses using a UPLC H-class (Waters), with an ACQUITY CSH C18 column (Waters) with dimensions 2.1 × 100 mm x 1.7 µm using a mobile phase:

Phase A) MilliQ water **Contributed by users** plus [M] **0.1 % (v/v)** Formic acid, LC-MS grade **Thermo Fisher Scientific Catalog #28905** .

Phase B) Acetonitrile **Contributed by users** .

25 Set the flow rate to 0.4 mL/min.

26 Apply the segmented gradient as follows:

A	B	C	D
Time (min)	Flow (mL/min)	A (%)	B (%)
Initial	0.400	90.0	10.0
2.00	0.400	90.0	10.0
7.00	0.400	10.0	90.0
9.00	0.400	10.0	90.0
11.00	0.400	90.0	10.0
13.00	0.400	90.0	10.0

27 Set the temperature to **30 °C** , while the injection volume must be **5 µL** for the positive and **2 µL** for the negative ionization modes.

MASS SPECTROMETRY ANALYSES

28 Perform the analyses using the XEVO-G2XSQTOF (Waters) instrument equipped with an electrospray ion source.

29 Use a [M]**0.5 millimolar (mM)** sodium formate solution for the instrument calibration.

30 Perform the analyses in the positive (+) and negative (-) ionization modes.

31 Optimize the source parameters for better performance. Suggestion of initial parameters:

A	B	C
Parameters	Positive Ion Mode	Negative Ion Mode
Source temperature (°C)	140	140
Desolvation temperature (°C)	550	550
Desolvation flow (L/h)	900	900
Capillary (kV)	3	2.5
Sampling cone (kV)	30	40
Cone gas flow (L/h)	10	50

32 Acquire the spectra under the acquisition range of 50-1200 Da, using the MS^E approach (6 V for low-energy, and a 15-30 V ramp for high-energy scanning).

33 Use Leucine encephalin [M]**200 Parts per Million (PPM)** in an Acetonitrile **Contributed by users** : MilliQ water **Contributed by users** 1:1 v/v) as the lockmass, infused at 25 µL/min.

DATA PROCESSING AND POTENTIAL IDENTIFICATION OF COMPOUNDS

34 Process the .RAW files obtained after LC-ESI-MS analysis by using the Progenesis QI software 2.4.69.11 (Nonlinear Dynamics, Newcastle, UK)). Choose to use centroid data and the mass resolution of 40,000. Perform peak alignment based on the QC samples. Select the following adduct species: [M+H]+, [M+Na]+, [M+K]+, [M+ACN+Na]+, [M+ACN+H]+, [M+H -H2O]+, [M+H-2H2O]+ for the positive ion mode, and [M-H]-, [M+FA-H]-, [M+Na-2H]-, [M-H2O-H]-, [M +Cl]-, for the negative ion mode. Progenesis QI generates a table containing the intensity of the ions listed according to their nominal masses for each sample. This software also generates MS^E -based putative identification of compounds.

35 Use the following databases to suggest the identifications : Lipid Maps (http://www.lipidmaps.org/), LipidBlast (https://fiehnlab.ucdavis.edu/projects/LipidBlast) and Human Metabolome Database (http://www.hmdb.ca/metabolites).

36 Use the following parameters for identification : mass error of the precursor ≤ 5 ppm, mass error of the fragment ≤ 10 ppm, mass precision, and isotopic similarity.

A	B	C	D	E	F	G	H	I	J
**Compound**	**Compound ID**	**Adducts**	**Formula**	**Score**	**Fragmentation Score**	**Mass Error (ppm)**	**Isotope Similarity**	**Description**	**Annotation Confidence Level***
Mode Negative									
4.58_245.0920m/z	HMDB0004259	M-H2O-H	C13H16N2O4	38,4	0	-4,29	96,9	Acetyl-N-formyl-5-methoxykynurenamine	3
8.13_301.2162m/z	HMDB0011134	M-H2O-H	C20H32O3	39,9	5,12	-3,30	98,4	5-HETE	2
9.00_305.2475m/z	HMDB0002925	M-H	C20H34O2	38,7	0	-3,67	97,8	8,11,14-Eicosatrienoic acid	3
8.59_329.2473m/z	HMDB0001976	M-H	C22H34O2	38,8	0	-4,08	98,8	Docosapentaenoic acid (22n-6)	3
8.49_303.2320m/z	HMDB0001043	M-H	C20H32O2	40,3	5,73	-3,27	99,6	Arachidonic acid	2
0.61_232.0824m/z	HMDB0012150	M+FA-H	C8H13NO4	40	11,7	-1,46	90,2	2-Keto-6-acetamidocaproate	2
0.61_134.0460m/z	HMDB0000056	M+FA-H	C3H7NO2	39,8	4,66	1,51	96,0	beta-Alanine	2
0.54_802.6697m/z	HMDB0013408	M-H	C46H94NO7P	37,1	0	0,29	85,9	PC(o-16:0/22:0)	3
1.67_291.0973m/z	HMDB0011741	M-H2O-H	C14H18N2O6	38,7	0	-4,25	98,5	gamma-Glutamyltyrosine	3
3.52_241.1184m/z	HMDB0011170	M-H2O-H	C11H20N2O5	41,1	13,4	-3,83	96,4	gamma-Glutamylisoleucine	2
0.75_151.0247m/z	HMDB0000139	M+FA-H	C3H6O4	39,5	3,17	-0,62	95,0	Glyceric acid	2
Mode Positive									
0.56_251.1008n	HMDB0000101	M+Na, M+K, M+H, M+H-H2O	C10H13N5O3	39	5,71	-4,30	94,5	Deoxyadenosine	2
0.54_139.0743n	HMDB0012234	M+H-H2O, M+H	C6H9N3O	39,6	10,2	-2,20	90,2	Histidinal	2
4.43_407.1214m/z	HMDB0062198	M+ACN+H	C12H19N3O8S	38,9	3,93	-4,58	96,0	2-S-glutathionyl acetate	2
1.39_298.0970m/z	HMDB0001173	M+H	C11H15N5O3S	43,5	19	0,59	98,9	5'-Methylthioadenosine	2
0.77_153.0402m/z	HMDB0000292	M+H	C5H4N4O2	39,6	4,75	-3,13	97,2	Xanthine	2
0.65_152.0566m/z	HMDB0000132	M+H	C5H5N5O	40,6	4,18	-0,46	99,4	Guanine	2

0.50_364.2445m/z	HMDB0011531	M+ACN+Na	C17H32O4	38,8	0,505	-4,36	98,6	MG(0:0/14:1(9Z)/0:0)	2

* Annotation Confidence Level. (1) Reference standard confirmed structure; (2) exact mass, isotopic pattern, retention time, and MS/MS spectrum matched to an in-house spectral database or literature spectra; (3) putative ID assignment based only on elemental formula match with exact mass and isotopic pattern, and (4) unknown compound.

STATISTICAL ANALYSES

37

The statistical analyses were performed using the Metaboanalyst web platform (version 5.0 - https://www.metaboanalyst.ca/). For the negative ionization mode: data was normalized by the IS (p-Fluoro-DL-phenylalanine **Sigma-aldrich Catalog # F525**), log-transformed, and Pareto scaled. The same procedure was used for the positive ionization mode, but the cubic transformation was applied instead of the log.

Perform multiple volcano plots to test between the compared culture conditions and also to blank samples.

38 Rank the relevant molecular features according to the false discovered ratio (FDR) and log2 fold change (FC) values.

39 For the assignment of cell secretomes, consider only the molecular features that present FDR value < 0.05 and log2 FC>0 when compared to the secretome of the culture medium (blank sample).
