# Supplementary material for: Liquid chromatography coupled to high-resolution mass spectrometry metabolomics: A useful tool for investigating tumor secretome based on a three-dimensional co-culture model
Source: PLoS One. 2022 Sep 21;17(9):e0274623. doi: 10.1371/journal.pone.0274623 (PMC9491614; doi:10.1371/journal.pone.0274623)
Supplement: Attachment — Submitted filename: Response to Reviewers.docx [file pone.0274623.s002.docx]

**Reviewer #1: The manuscript by Pelosi et al. describes a protocol for 3D cell culture and its secretome analysis by liquid chromatography-mass spectrometry based-metabolomics. Overall, the manuscript is easy to follow and provides a protocol on a recent field of study. There is a growing interest in 3D cell culture metabolomics in the literature. However, the LC-MS based-metabolomics section of the protocol is poorly described, missing various important information. Because of this and other issues pointed out below, I recommend a revision of the manuscript.**

We thank the reviewer for the positive evaluation of our manuscript. The suggestions brought during this revision helped us improve the manuscript's quality. We hope we have fully addressed the concerns and questions raised here, all suggestions were considered and we are ready to review any further points that might become necessary.

**Title**

**Please, use "liquid Chromatography coupled to high-resolution mass spectrometry" instead of "High resolution liquid chromatography coupled to high resolution mass Spectrometry" (also check other manuscript sections since there is a lack of formatting) | cannot see any reason to use "High resolution liquid chromatography" since metabolomics applications are mainly performed using columns with sub-2 um particles.**

Answer: The reviewer is correct. We have changed the title as suggested and revised the manuscript to exclude the use of "high-resolution liquid chromatography."

New title: Liquid chromatography coupled to high-resolution mass spectrometry metabolomics: A useful tool for investigating tumor secretome based on a three-dimensional co-culture model

**Abstract**

**Correct "high-performance liquid-chromatography coupled with high resolution mass spectrometry" to "liquid chromatography coupled to high-resolution mass spectrometry"**

Answer: The reviewer is right. We have changed the abstract as suggested and revised the manuscript to exclude the use of "high-performance liquid-chromatography coupled with high-resolution mass spectrometry."

New […] We present an untargeted metabolomic protocol based on the hybrid technique of liquid chromatography coupled with high-resolution mass spectrometry for the analysis of low-molecular-weight metabolites released into the culture medium by 3D cultures and co-culture (secretome model). […]

**Introduction**

**The advantages of using the secretome instead of intracellular metabolites of isolated cells for metabolomics are stated, But what are the disadvantages/limitations of this approach? The authors need to provide some thoughts on it.**

Answer: We thank the reviewer for highlighting it. We have rephrased the lines and added the highlighted information, as required.

[…] This approach has the following advantages: ensuring little (or non-existent) handling of cells, which avoids the production of artifacts, allowing the monitoring of metabolic activity in response to experimental disturbances without cell disruption, enabling the monitoring of metabolic changes over time within the same culture and avoid carrying out long and multiple extraction procedures, which also enable the production of technical artifacts that can lead to concealment or unwanted manipulation of biological results [25]. However, the disadvantages of working with the secretome include matrix effects related to the salty media composition, which is rich in sugars, lipids, proteins, and water and might interfere in the analysis. The dilution of the metabolites of interest in the media also impacts the detection sensitivity [26].

**Material and methods (protocols.o)**

**- Step 20: specify how the culture medium is collected from the plate, And please confirm If it is 50 mg of IPA or if It should be 50 uL.**

Answer: The following changes were provided.

Text excluded: Add an aliquot of iced isopropanol 50 mg to the culture medium 200 µL and maintain

Overnight at -20 °C, for secretome analysis.

Text included:

Step 20: Using a pipet, take 200 µL of the culture medium of each well and place it into a microtube. Then, add 50 µL of iced isopropanol and keep the microtube at -20 °C overnight. Prepare each sample in triplicate. Additionally, prepare pooled quality control (QC) samples. For that, an aliquot (20 µL) of each sample must be collected and pooled together in a microtube to generate a quality control (QC) sample containing the chemical composition representative of the sample set. Homogenize this sample and split it into different vials, then prepare them following the same extraction protocol. Use QC samples for system suitability before batch analysis. Also, inject a QC sample after 10 samples to investigate and filter instrumental variation. Blank samples (N=3) must also be prepared using the culture medium without adding any cell or culture and following the complete protocol for sample preparation. Samples must be organized by starting with QC samples, followed by blank samples, and then the set of test samples randomly organized. A QC sample must be placed every 10 samples. The final sample must be a QC sample.

**- Step 21: use G force (ST uni) instead of RPM.**

Answer: Changes provided accordingly.

Text excluded: Centrifuge the samples for 12500 rpm, 4°C, 10 minutes.

Text included: Centrifuge the samples for 12880 x g, 4°C, 10 minutes.

**- Step 23: specify which "fluoride-phenylalanine" standard was used, its source/catalog number, and solvent.**

Answer: The following information was added:

Text excluded: Resuspend dry extracts in 150 µL of an internal fluoride-phenylalanine standard solution at 200 Micromolar (µM).

Text included: Resuspend the dry extracts in 150 µL of a solution composed of p-Fluoro-DL-phenylalanineSigma-aldrichCatalog # F525 at 200 micromolar (µM) in MethanolSigma AldrichCatalog #M3641 and MilliQ water (1:1 v/v)

**- Step 24: confirm if the formic acid was indeed not added to acetonitrile.**

Answer: Formic acid was only added to mobile phase A.

**- Step 27: What are the reasons for such a low column over temperature (20 °C)?**

Answer: We apologize for the mistake. The correct temperature is 30 °C. The text was revised accordingly.

Text excluded: Set the temperature at 20 °C, while the injection volume is 5 μL for positive and 2 μL for negative modes.

Text included: Set the temperature at 30 °C, while the injection volume is 5 μL for positive and 2 μL for negative modes.

**- Step 34: detail the parameters used for RAW data processing in Propeness IQ software**

Answer: The following changes were provided

Text excluded: Process the raw data with Progenesis IQ (Waters) software for peak detection, alignment, integration, deconvolution, data filtering, ion annotation, and MSE based putative identification of compounds

Text included: Process the .RAW files obtained after LC-ESI-MS analysis by using the Progenesis QI software 2.4.69.11 (Nonlinear Dynamics, Newcastle, UK)). Choose to use centroid data and the mass resolution of 40,000. Perform peak alignment based on the QC samples. Select the following adduct species: [M+H]+, [M+Na]+, [M+K]+, [M+ACN+Na]+, [M+ACN+H]+, [M+H -H2O]+, [M+H-2H2O]+ for the positive ion mode, and [M-H]-, [M+FA-H]-, [M+Na-2H]-, [M-H2O-H]-, [M +Cl]-, for the negative ion mode. Progenesis QI generates a table containing the intensity of the ions listed according to their nominal masses for each sample. This software also generates MS^E^ -based putative identification of compounds.

**- Step 36: what were the MS/MS and isotopic similar scores employed?**

Answer: A table containing the fragmentation and isotope similarity scores was provided in protocols as follows:

| **Compound** | **Compound ID** | **Adducts** | **Formula** | **Score** | **Fragmentation Score** | **Mass Error (ppm)** | **Isotope Similarity** | **Description** | **Annotation Level*** |
| --- | --- | --- | --- | --- | --- | --- | --- | --- | --- |
| **Mode Negative** |  |  |  |  |  |  |  |  |  |
| 4.58_245.0920m/z | HMDB0004259 | M-H2O-H | C13H16N2O4 | 38,4 | 0 | -4,29 | 96,9 | Acetyl-N-formyl-5-methoxykynurenamine | 3 |
| 8.13_301.2162m/z | HMDB0011134 | M-H2O-H | C20H32O3 | 39,9 | 5,12 | -3,3 | 98,4 | 5-HETE | 2 |
| 9.00_305.2475m/z | HMDB0002925 | M-H | C20H34O2 | 38,7 | 0 | -3,67 | 97,8 | 8,11,14-Eicosatrienoic acid | 3 |
| 8.59_329.2473m/z | HMDB0001976 | M-H | C22H34O2 | 38,8 | 0 | -4,08 | 98,8 | Docosapentaenoic acid (22n-6) | 3 |
| 8.49_303.2320m/z | HMDB0001043 | M-H | C20H32O2 | 40,3 | 5,73 | -3,27 | 99,6 | Arachidonic acid | 2 |
| 0.61_232.0824m/z | HMDB0012150 | M+FA-H | C8H13NO4 | 40 | 11,7 | -1,46 | 90,2 | 2-Keto-6-acetamidocaproate | 2 |
| 0.61_134.0460m/z | HMDB0000056 | M+FA-H | C3H7NO2 | 39,8 | 4,66 | 1,51 | 96 | beta-Alanine | 2 |
| 0.54_802.6697m/z | HMDB0013408 | M-H | C46H94NO7P | 37,1 | 0 | 0,29 | 85,9 | PC(o-16:0/22:0) | 3 |
| 1.67_291.0973m/z | HMDB0011741 | M-H2O-H | C14H18N2O6 | 38,7 | 0 | -4,25 | 98,5 | gamma-Glutamyltyrosine | 3 |
| 3.52_241.1184m/z | HMDB0011170 | M-H2O-H | C11H20N2O5 | 41,1 | 13,4 | -3,83 | 96,4 | gamma-Glutamylisoleucine | 2 |
| 0.75_151.0247m/z | HMDB0000139 | M+FA-H | C3H6O4 | 39,5 | 3,17 | -0,62 | 95 | Glyceric acid | 2 |
| **Mode Positive** |  |  |  |  |  |  |  |  |  |
| 0.56_251.1008n | HMDB0000101 | M+Na, M+K, M+H, M+H-H2O | C10H13N5O3 | 39 | 5,71 | -4,3 | 94,5 | Deoxyadenosine | 2 |
| 0.54_139.0743n | HMDB0012234 | M+H-H2O, M+H | C6H9N3O | 39,6 | 10,2 | -2,2 | 90,2 | Histidinal | 2 |
| 4.43_407.1214m/z | HMDB0062198 | M+ACN+H | C12H19N3O8S | 38,9 | 3,93 | -4,58 | 96 | 2-S-glutathionyl acetate | 2 |
| 1.39_298.0970m/z | HMDB0001173 | M+H | C11H15N5O3S | 43,5 | 19 | 0,59 | 98,9 | 5'-Methylthioadenosine | 2 |
| 0.77_153.0402m/z | HMDB0000292 | M+H | C5H4N4O2 | 39,6 | 4,75 | -3,13 | 97,2 | Xanthine | 2 |
| 0.65_152.0566m/z | HMDB0000132 | M+H | C5H5N5O | 40,6 | 4,18 | -0,46 | 99,4 | Guanine | 2 |
| 0.50_364.2445m/z | HMDB0011531 | M+ACN+Na | C17H32O4 | 38,8 | 0,505 | -4,36 | 98,6 | MG(0:0/14:1(9Z)/0:0) | 2 |

* Annotation Confidence Level. (1) Reference standard confirms structure (2) exact mass, isotopic pattern, retention time, and MS/MS spectrum matched to an in-house spectral database or literature spectra (3) putative ID assignment based only on elemental formula match with exact mass and isotopic pattern. (4) unknown compound.

**- Step 37 details how data is pre-processed (scaling, normalization, et).**

Answer: The following changes were provided

Text included: The statistical analyses were performed using the Metaboanalyst web platform (version 5.0 - <https://www.metaboanalyst.ca/>). For the negative ionization mode: data was normalized by the IS (p-Fluoro-DL-phenylalanineSigma-aldrichCatalog # F525 ), log-transformed, and Pareto scaling was applied. The same procedure was used for the positive ionization mode, but the cubic transformation was applied instead of the log.

**- Information about quality control (pooled QC, Standard mix solution) and blanks (extraction blanks, solvent blanks) are missing**

Answer: The information was added in step 20, as previously mentioned.

**- Information about the number of samples investigated and the replicates are missing**

Answer: The information was added in step 20, as previously described.

**Expected results**

**It is unclear why the authors have chosen to co-culture HT-29 human colon carcinoma cells with 3T3-L1 preadipocytes. An explanation is missing in the manuscript**

Answer: We thank the reviewer for pointing this out. We included the following text:

The presence of adipose tissue can influence the development of CRC *in vivo* [27]. To better understand this influence *in vitro*, we used a model of HT-29 cell line as cancer cells and 3T3-L1 as adipocytes[28]. These cell lines were cultured separately and co-cultured, and their secretome was used to investigate the tumor microenvironment and the interaction between cancer cells and the adipocyte tissue.

**Please,Inform in Table 1 that the molecular features refer to comparison with a blank sample.**

Answer: Information provided, as requested.

Table 1. Molecular features of 3D cultured cells^a^

| **Cell type** | **NEGATIVE MODE** | **POSITIVE MODE** |
| --- | --- | --- |
| HT | 191 features | 3 features |
| 3T3 | 329 features | 267 features |
| HT + 3T3 | 129 features | 14 features |

a. characteristic metabolites of each spheroid after comparison with the blank samples (cultured media only)

**An explanation about why choosing the CSH C18 column to perform the secretome analysis must be provided since column Chemistry greatly impacts the class of compounds detected.**

Answer: We thank the reviewer for highlighting such a need. Information was added as requested.

CSH (charged surface hybrid) particles were designed to enable sample loading and improve peak symmetry when using low ionic strength mobile phases, as instructed by the manufacturer. Reversed-phase columns, such as the chosen CSH C18, are broadly used for metabolomics investigation in different matrices such as plasma [29], serum [30], urine [31], tissue [32], and cell cultures [33], as chromatographic columns of this type generally result in the detection of more features [34].

**There is a Considerable discrepancy between the number of features detected and metabolites annotated in Table 2. So, a brief discussion should be provided about it.**

Answer: We thank the reviewer for such a comment. We provided the information as follows:

Although Table 2 brings a small number of metabolites when compared to the detected ones, these metabolites were the ones that met the statistical criteria of relevance, as well as the annotation criteria based on MS/MS and isotopic pattern recognition. Indeed, in untargeted metabolomics, linking chemical structures to the data obtained by mass spectrometry remains a significant challenge. The vast majority of information collected by metabolomics is the so-called "dark matter," i.e., chemical signatures that remain uncharacterized [35].

**Why is there m/z after the feature code (5.49_303.2320m/z) in table 2?**

Answer: The acronym "m/z" is part of the Progenesis naming process. Features can return labeled with "m/z," referring to an ion, or with "n," referring to a neutral molecule also matched with the adduct species of the suggested compound.

**The correct representation of retention time is tR (R subscript) and not RT.**

Answer: Changes provided accordingly.

**Raw data files should be made available on the data repository (e.g., Metabolomics Workbench and MetaboLights).**

Answer: The project was submitted on the metabolights platform under the code MTBLS5375.

**New References**

26. Pinu FR, Villas-Boas SG. Extracellular Microbial Metabolomics: The State of the Art. Metabolites. 2017;7(3).

27. Jochem C, Leitzmann M. Obesity and Colorectal Cancer. Recent Results Cancer Res. 2016;208:17-41.

28. Zebisch K, Voigt V, Wabitsch M, Brandsch M. Protocol for effective differentiation of 3T3-L1 cells to adipocytes. Anal Biochem. 2012;425(1):88-90.

29.	Silva AAR, Cardoso MR, Rezende LM, Lin JQ, Guimaraes F, Silva GRP, et al. Multiplatform Investigation of Plasma and Tissue Lipid Signatures of Breast Cancer Using Mass Spectrometry Tools. Int J Mol Sci. 2020;21(10).

30. Zardini Buzatto A, Tatlay J, Bajwa B, Mung D, Camicioli R, Dixon RA, et al. Comprehensive Serum Lipidomics for Detecting Incipient Dementia in Parkinson's Disease. J Proteome Res. 2021;20(8):4053-67.

31. Struck-Lewicka W, Kordalewska M, Bujak R, Yumba Mpanga A, Markuszewski M, Jacyna J, et al. Urine metabolic fingerprinting using LC-MS and GC-MS reveals metabolite changes in prostate cancer: A pilot study. J Pharm Biomed Anal. 2015;111:351-61.

32. Al-Sari N, Suvitaival T, Mattila I, Ali A, Ahonen L, Trost K, et al. Lipidomics of human adipose tissue reveals diversity between body areas. PLoS One. 2020;15(6):e0228521.

33. Ali AS, Raju R, Ray S, Kshirsagar R, Gilbert A, Zang L, et al. Lipidomics of CHO Cell Bioprocessing: Relation to Cell Growth and Specific Productivity of a Monoclonal Antibody. Biotechnol J. 2018;13(10):e1700745.

34. Yanes O, Tautenhahn R, Patti GJ, Siuzdak G. Expanding coverage of the metabolome for global metabolite profiling. Anal Chem. 2011;83(6):2152-61.

35. Silva RR, Dorrestein PC, Quinn RA. Illuminating the dark matter in metabolomics. Proc Natl Acad Sci U S A. 2015;112(41):12549-50.
